# Extrinsic motivation and student engagement in higher education: the mediating role of basic psychological need satisfaction across instructional modalities

**DOI:** 10.3389/fpsyg.2026.1962667

**Published:** 2026-10-26

**Authors:** Xingquan Xie, Ying Hu, Shuang Wang

**Affiliations:** 1 School of Marxism, Chengdu University of Technology, Chengdu, China; 2 West China Biomedical Big Data Center, West China Hospital, Sichuan University, Chengdu, China

**Keywords:** extrinsic motivation, basic psychological need satisfaction, student engagement, instructional modalities, self-determination theory, higher education

## Abstract

**Introduction:**

Extrinsic motivation is pervasive in higher education, yet whether it sustains student engagement through basic psychological need satisfaction across instructional modalities remains unclear. Drawing on self-determination theory (SDT), this study examined the mediated pathway from extrinsic motivation to student engagement via need satisfaction and compared this pathway across face-to-face, online, and blended instruction.

**Methods:**

Survey data came from 943 undergraduates in nine intact classes assigned to face-to-face (*n* = 394), online (*n* = 256), and blended (*n* = 293) conditions. We analyzed the data with multi-group structural equation modeling, including measurement invariance tests and bootstrap mediation based on 5,000 resamples.

**Results:**

Extrinsic motivation positively predicted students’ basic psychological need satisfaction (β = 0.56 -0.58, *p* < .01), which in turn predicted student engagement (β = 0.54 -0.55, *p* < .01). Need satisfaction significantly mediated the motivation -engagement relationship in all modalities, with the indirect effect accounting for 55 -71% of the total effect; the direct effect was nonsignificant only in the online group.

**Discussion:**

Extrinsic motivation sustains student engagement chiefly when internalized through students’ basic psychological need satisfaction, and fully online instruction is particularly dependent on this mechanism. The findings extend SDT-based accounts of student engagement and guide the design of need-supportive, AI-enhanced higher education.

## 1 Introduction

Student engagement is widely regarded as a central marker of successful higher education, predicting academic achievement, retention and wellbeing across face-to-face, blended and online learning contexts (Fredricks et al., 2004; Reeve et al., 2025; Wong and Liem, 2022). As digital and AI-supported learning become increasingly common in universities, understanding how instructional arrangements motivate students to invest and remain engaged has become a particularly pressing concern for both researchers and practitioners (Chiu et al., 2024; Xu and Zhi, 2026). Self-determination theory (SDT) offers a useful framework for this question, because it distinguishes among four regulations of extrinsic motivation—external, introjected, identified and integrated—and locates sustained engagement in the satisfaction of three innate psychological needs: autonomy, competence and relatedness (Ryan and Deci, 2000a; Ryan and Deci, 2020). Yet the motivational consequences of rewards remain contested. Meta-analytic evidence shows that expected, tangible rewards tend to undermine intrinsic motivation, whereas recognition and non-controlling feedback tend to sustain it, indicating that rewards either frustrate or satisfy psychological needs depending on how they are delivered (Deci et al., 1999; Malek et al., 2020). Whether this mechanism operates similarly across instructional modalities is not self-evident. In face-to-face settings, dense social presence readily supports relatedness and competence; in fully online settings, these needs must be deliberately engineered through digital tools; and in blended settings, students move between the two, so need satisfaction depends on how seamlessly teacher and digital support are integrated (Chiu, 2022; Raes et al., 2019; van der Rijst et al., 2023). Although prior work has linked teacher and digital support to engagement through motivational and self-regulatory processes (Kok et al., 2025; Liu et al., 2023), few studies have simultaneously tested whether basic psychological need satisfaction mediates the relation between extrinsic motivation and engagement and compared this mediation across face-to-face, blended and online groups within a single higher-education sample. The present study addresses this gap by modelling extrinsic motivation, basic psychological need satisfaction and student engagement with multi-group structural equation modelling, thereby clarifying how extrinsic rewards and feedback should be designed in each modality to support rather than frustrate students’ psychological needs.

## 2 Literature review

### 2.1 Extrinsic motivation and basic psychological need satisfaction across instructional modalities

Self-determination theory (SDT) conceptualises extrinsic motivation not as a single undifferentiated force but as a continuum of four regulations that vary in their degree of internalisation: external regulation, introjected regulation, identified regulation, and integrated regulation (Ryan and Deci, 2000a). External regulation, the least autonomous form, describes behaviour driven by external contingencies such as rewards or punishments; introjected regulation is internal but still controlling, because students act to avoid guilt or to protect contingent self-esteem; identified regulation reflects personally endorsed goals; and integrated regulation, the most autonomous form of extrinsic motivation, is congruent with the self (Ryan and Deci, 2000a). Early behaviourist accounts treated extrinsic rewards as inherently controlling, yet meta-analytic evidence shows that their motivational consequences depend on how they are experienced: expected, tangible rewards tend to undermine intrinsic motivation, whereas positive, non-controlling feedback tends to sustain it (Deci et al., 1999). Subsequent work refined this picture by distinguishing reward types, demonstrating that financial rewards are negatively related to intrinsic task motivation while recognition and social rewards are positively related, precisely because the latter convey competence and acknowledge personal value (Malek et al., 2020). Tangible and intangible rewards also operate through situational extrinsic motivation, indicating that the internalisation of regulation is itself sensitive to the reward structure and its social framing (Yoon et al., 2015). Within SDT, these dynamics are anchored in three innate psychological needs—autonomy, competence and relatedness—and rewards perceived as controlling frustrate them, whereas rewards that provide autonomy support, structure and involvement satisfy them (Ryan and Deci, 2020). Whether instructional modality makes a difference is therefore a question about the social context in which rewards are delivered. Face-to-face instruction centres on direct, instructor-led teaching in a physical classroom; online instruction delivers the curriculum through digital platforms with limited physical co-presence; and blended instruction integrates scheduled face-to-face sessions with online activities, combining the strengths of each (Garrison and Kanuka, 2004; Dziuban et al., 2018; Boelens et al., 2017). In face-to-face settings, immediate teacher feedback and peer interaction readily satisfy relatedness and competence; in fully online settings, the same feedback must be deliberately engineered through digital tools, and relatedness is more easily thwarted; in blended settings, students move between the two contexts, so need satisfaction depends on how seamlessly teacher and digital support are integrated across modes (Chiu, 2022; Raes et al., 2020; Bower, 2015). Direct comparisons of how the four regulations map onto need satisfaction across face-to-face, blended and online learning nevertheless remain scarce, especially in higher education.

### 2.2 Basic psychological need satisfaction and student engagement in AI-enhanced higher education

Student engagement is widely treated as a multidimensional construct that combines behavioural, emotional, cognitive and agentic investment in learning, and it is now established that these dimensions serve distinct functional purposes rather than acting as interchangeable indicators of a single latent state (Fredricks et al., 2004; Reeve et al., 2025; Wong and Liem, 2022). Behavioural engagement refers to participation and persistence, emotional engagement to affective reactions to teachers and peers, cognitive engagement to strategic and deep learning, and agentic engagement to students’ proactive contribution to the instructional process (Reeve et al., 2025). Because engagement is energised rather than automatic, SDT locates its proximal antecedent in the satisfaction of basic psychological needs. SDT is preferred to alternative need or motivation frameworks because it specifies a small, universal set of innate psychological needs—autonomy, competence and relatedness—whose satisfaction is held to be necessary for optimal functioning across contexts, and it therefore offers a parsimonious, cross-contextual mechanism for linking instructional environments to engagement (Deci and Ryan, 2008; Niemiec and Ryan, 2009). When learners experience autonomy, competence and relatedness, they invest more voluntarily, more deeply and more durably in academic tasks (Ryan and Deci, 2000b; Ryan and Deci, 2020). In online learning during the COVID-19 disruption, need satisfaction was shown to predict greater student engagement, with relatedness support emerging as especially decisive (Chiu, 2022). This link has become particularly consequential in AI-enhanced higher education, where teacher support and digital or AI support jointly shape how competent and autonomous students feel; studies of AI-based chatbots and AI-assisted learning report that perceived support for these needs translates directly into motivated learning and sustained engagement (Chiu et al., 2024; Xu and Zhi, 2026), and meta-analytic evidence indicates that AI chatbots can improve learning outcomes when they scaffold rather than replace self-regulation (Wu and Yu, 2024). Reviews of AI in teaching and learning similarly note that educational technology is not inherently motivating but becomes so when it scaffolds competence and preserves student autonomy (Ng et al., 2023). In blended learning specifically, teacher and digital support relate to satisfaction through student engagement, confirming that engagement sits between the instructional environment and valued outcomes (Kok et al., 2025; Raes et al., 2019). At the same time, the definition and measurement of engagement remain contested, so it should be distinguished analytically from motivation and achievement rather than collapsed with either (Sinatra et al., 2015).

### 2.3 Basic psychological need satisfaction as a mediating mechanism

If need satisfaction is the proximal driver of engagement, and if extrinsic rewards shape how students internalise regulation, then basic psychological need satisfaction should logically function as the mediating mechanism that connects external regulation to sustained investment rather than as a parallel outcome. This mediational logic is consistent with accumulating evidence that motivational and contextual effects on engagement operate indirectly through psychological needs and related self-regulatory processes: teacher support predicts engagement through self-efficacy and achievement goal orientation in EFL learners (Liu et al., 2023), teacher support predicts academic engagement through motivated learning behaviour (Li et al., 2024), and teacher and digital support predict learner satisfaction through engagement itself in blended higher education (Kok et al., 2025). Flow experiences in AI-assisted learning further illustrate that engagement emerges when AI and teacher support jointly satisfy the conditions for optimal challenge, competence and autonomy (Xu and Zhi, 2026). What remains unresolved is whether the strength of this mediation differs across instructional modalities. On theoretical grounds, it should. In face-to-face settings, dense social presence readily satisfies relatedness, strengthening the indirect path; in fully online settings, relatedness is harder to establish and autonomy support must be deliberately designed, so controlling rewards may attenuate the indirect effect (Chiu, 2022; Raes et al., 2020). Blended settings occupy an intermediate position, where the mediated pathway is strengthened when teacher and digital support are well integrated but weakened when they are not (van der Rijst et al., 2023; Raes and Depaepe, 2020). HyFlex models likewise suggest that flexibility is beneficial only when accompanied by coherent pedagogy rather than mere technological access (Howell, 2022). Yet no study has simultaneously estimated the indirect extrinsic-motivation-to-engagement path through need satisfaction and formally compared it across face-to-face, blended and online groups within a single higher-education sample.

### 2.4 The present study and research aims

Despite this progress, three gaps remain. First, most prior work treats extrinsic motivation as a unidimensional predictor or conflates intrinsic motivation with basic need satisfaction, thereby obscuring the distinct OIT regulations and the precise psychological needs they serve (Ryan and Deci, 2000a; Ryan and Deci, 2020). Second, although rewards and need satisfaction have each been linked to engagement, the indirect pathway that runs from extrinsic regulations through basic psychological need satisfaction to engagement has rarely been tested with a measurement model that separates the three constructs and reports convergent and discriminant validity (Deci et al., 1999; Malek et al., 2020). Third, even fewer studies compare this mediational mechanism across face-to-face, blended and online learning within the same design, so it is unclear whether internalisation proceeds similarly across modalities or whether instructional context qualifies the effect (Chiu, 2022; Raes et al., 2020). This gap is especially salient in AI-enhanced higher education, where digital and teacher support are increasingly combined but their motivational consequences across modes remain poorly understood (Chiu et al., 2024; Xu and Zhi, 2026). The present study therefore examines how extrinsic motivation—operationalised through external, introjected, identified and integrated regulation—is associated with student engagement in higher education, and whether this association operates through basic psychological need satisfaction, while comparing the structure of the relationships across face-to-face, blended and online instructional modalities. A multi-group structural equation model with bootstrap mediation tests is used to determine whether the indirect path is invariant across modes or differs in strength, and the findings are intended to clarify how rewards and digital feedback should be designed in each modality to support rather than frustrate students’ basic psychological needs. Drawing on self-determination theory (Ryan and Deci, 2000b), the following hypotheses were proposed:

*H1*: Extrinsic motivation is positively associated with basic psychological need satisfaction.

*H2*: Basic psychological need satisfaction is positively associated with student engagement.

*H3*: Basic psychological need satisfaction mediates the relationship between extrinsic motivation and student engagement, such that extrinsic motivation predicts student engagement both directly and indirectly through basic psychological need satisfaction.

## 3 Methods

### 3.1 Participants

The sample size comfortably exceeded the minimum recommended for covariance structure modelling (MacCallum et al., 1996). Participants were drawn from two university-wide general-education courses, Outline of Chinese Modern History (a compulsory public course) and History of the People’s Republic of China (an elective public course), both designated by the university as smart courses delivered through the Xueyin Online platform. Combining convenience and quota sampling, questionnaires were administered both online and in person to first and second students enrolled in these courses, who represented a broad range of undergraduate majors (Table 1). Eligibility required prior experience in using either the Chaoxing Xuexitong mobile application or the desktop-based Xueyin Online smart-course platform for coursework in public courses. All All participants completed a Chinese-language questionnaire that provided pre-test and post-test measures of student engagement, and they gave written informed consent before taking part. The study adhered to ethical principles for questionnaire-based research (Nguyen et al., 2023).

**Table 1:** Demographic characteristics of the sample (*N* = 943).

Variable	Value	Description	Frequency
Hukou	1/0	Urban/Rural	68%/32%
Ethnicity	1/0	Han/Ethnic minority	94%/6%
Gender	1/0	Male/Female	59%/41%
Major	1/0	Science and engineering/Humanities and social sciences	73%/27%
Only-child status	1/0	Only child/Non-only child	64%/36%
Region	1–6	Southwest 34%; Northwest 10%; Northeast 15%; North 12%; Central 18%; Eastern coastal 11%	–
Teaching group	1	Face-to-face	41.78% (*n* = 394)
	2	Online	27.15% (*n* = 256)
	3	Blended	31.07% (*n* = 293)

### 3.2 Research design and procedure

This study used a quantitative, quasi-experimental design with a pre-test and three parallel instructional conditions (Kitchenham and Brereton, 2013). To establish baseline equivalence, a pre-test of student engagement was first administered to students enrolled in the selected courses, and between-group differences were tested; only classes that showed no significant pre-test differences were retained in the sampling frame. Nine intact classes were then selected, three for each of the three instructional modalities—(i) traditional face-to-face instruction, (ii) online teaching, and (iii) blended teaching. Because students remained in their enrolled classes rather than being individually randomised, the design is quasi-experimental; the instructors (rather than the students) were randomly assigned to the three conditions, and the classes were verified to be equivalent at pre-test. Of the final respondents, 41.78% belonged to the face-to-face group, 27.15% to the online group and 31.07% to the blended group. Screening proceeded in two stages—class-level selection based on pre-test equivalence, followed by respondent-level data cleaning (removal of missing, invalid, duplicate and extreme-value responses)—which yielded the final sample of 943 students. The research procedure is shown in Figure 1.

**Figure 1:**
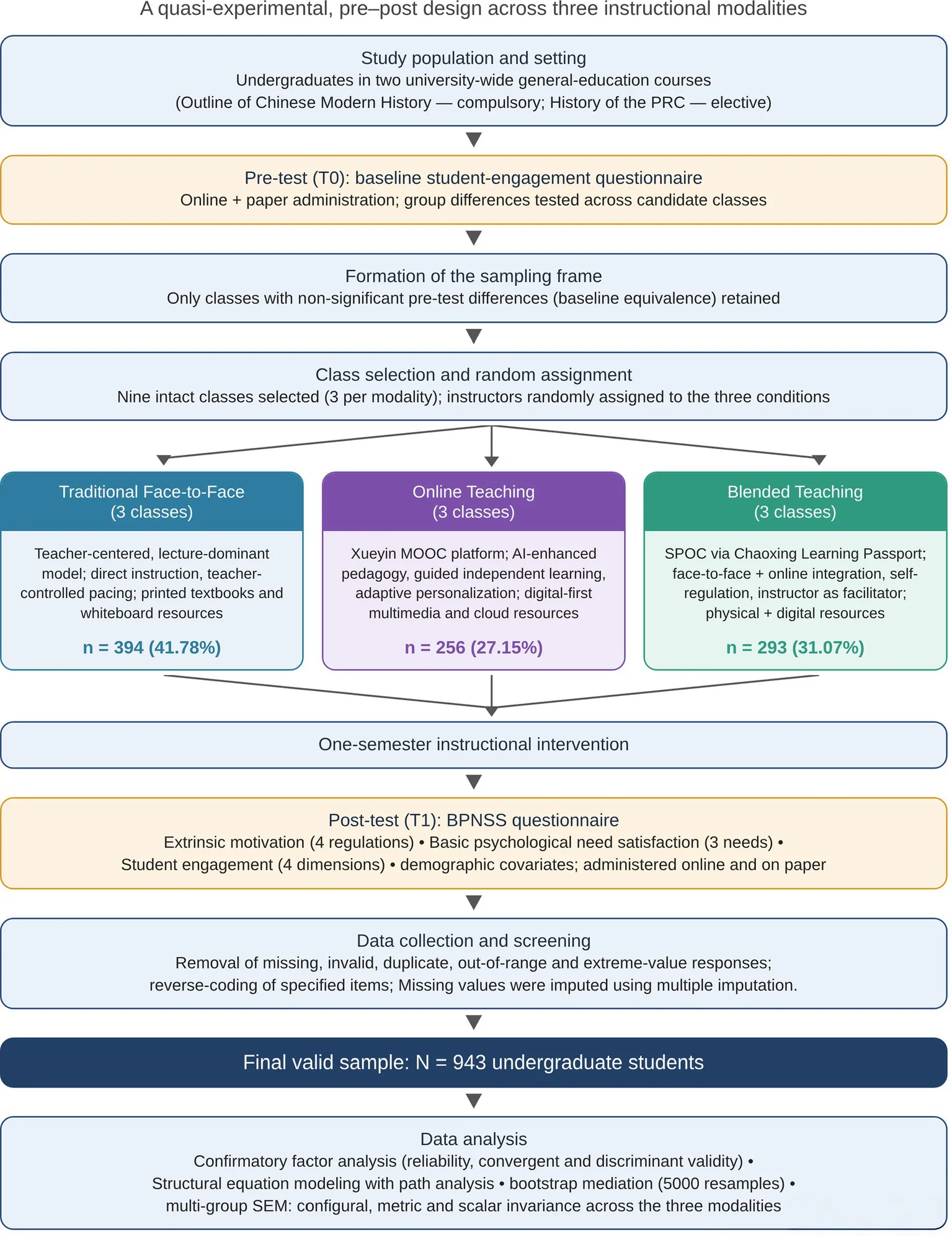
Research procedure.

### 3.3 Measures

The questionnaire combined the Self-Regulation Questionnaire–Academic (SRQ-A), the Intrinsic Motivation Inventory (IMI) and the Basic Psychological Need Satisfaction Scale (BPNSS). It operationalised 11 latent indicators across three constructs: extrinsic motivation (four regulatory dimensions), basic psychological need satisfaction (autonomy, competence and relatedness), and student engagement (four dimensions), together with core demographic covariates used as control variables. The instrument comprised 11 subscales, each containing five Likert-type items rated on a five-point scale (1 = strongly disagree to 5 = strongly agree). Content validity was established through expert appraisal, in which nine practising educators evaluated item wording for semantic clarity, linguistic appropriateness and contextual comprehensibility.

#### 3.3.1 Extrinsic motivation

Within the Organismic Integration Theory (OIT) sub-theory of SDT, the degree of self-determination is arranged along a continuum from low to high: external regulation → introjected regulation → identified regulation → integrated regulation, with intrinsic motivation lying further to the right. Ryan and Deci (2000a) define these as the varied types of extrinsic motivation and emphasise that extrinsic motivation is not a single construct but a set of motivational types that vary widely in relative autonomy. The extrinsic motivation measures were adapted from two foundational instruments—the SRQ-A (Ryan and Connell, 1989) and the IMI (McAuley et al., 1989). Extrinsic motivation was operationalised into four dimensions ordered by degree of autonomy: external regulation (behaviour driven by external contingencies), introjected regulation (internally pressured actions), identified regulation (personally endorsed goals), and integrated regulation (identity-congruent behaviours). Each indicator was measured with five Likert-scale items contextualised for undergraduate general-education settings. Illustrative items were: “I participate in discussion boards to avoid losing points or falling behind” (external regulation); “I try hard in the blended course to prove that I am capable” (introjected regulation); “I engage in this course because it aligns with my academic goals” (identified regulation); and “I apply myself to this course because what I learn has become part of who I am” (integrated regulation). Cognitive interviews established item clarity and contextual relevance (Patall et al., 2019). As shown in Table 2, confirmatory factor analysis and internal-consistency reliability (Cronbach’s *α* = 0.891) supported the measure.

**Table 2:** Confirmatory factor analysis and internal consistency reliability.

Latent variable	Indicator	Loading	SMC	CR	AVE	*α*
Extrinsic motivation	External regulation	0.834	0.506	0.925	0.754	0.891
	Introjected regulation	0.878	0.620			
	Identified regulation	0.895	0.645			
	Integrated regulation	0.865	0.577			
BPNS	Relatedness	0.754	0.546	0.920	0.793	0.869
	Competence	0.715	0.592			
	Autonomy	0.747	0.554			
Student engagement	Behavioral	0.844	0.520	0.914	0.727	0.874
	Affective	0.865	0.601			
	Cognitive	0.843	0.552			
	Agentic	0.831	0.572			

KMO = 0.816 (extrinsic motivation), 0.701 (BPNS), 0.809 (student engagement); Bartlett’s test *p* < 0.01 for all constructs.

#### 3.3.2 Instructional modalities

The role and function of the teacher differed markedly across the three modalities, consistent with comparative work on instructor roles in online and blended settings (Hung and Chou, 2015). The Traditional Face-to-Face Instruction Group implemented a teacher-centred, lecture-dominant model, with the instructor as the central authority, physical classroom arrangements (rows facing the board), direct instruction, teacher-assigned tasks and teacher-controlled pacing; resources were exclusively printed textbooks, handouts, worksheets and the whiteboard, consistent with pre-smart-education pedagogies (Zhu et al., 2016b). The Online Teaching Group used a digitally supported model centred on the Xueyin Online Massive Open Online Course (MOOC) platform, leveraging cloud-based infrastructure for scalable delivery (Al Tayeb et al., 2013); it emphasised AI-enhanced pedagogy (Barakina et al., 2021), technology-mediated interaction (All et al., 2016), guided independent learning and adaptive personalisation (Peng et al., 2019), with digital-first resources including multimedia content, e-textbooks and dynamic cloud tools (Mohammed and Zebaree, 2021). The Blended Teaching Group combined face-to-face sessions with online activities through Small Private Online Courses (SPOC) on the Chaoxing Learning Passport mobile platform, exemplifying smart-education ecosystems (Zhu et al., 2016a). Blended teaching is defined by this integration of—rather than a separate teacher-support variable within—in-person and online components; it emphasised proactive preparation, self-regulation, personalisation and instructor facilitation (Leem and Sung, 2019), and combined physical and digital materials to enrich peer interaction (Shu and Gu, 2023).

#### 3.3.3 Basic psychological need satisfaction

Basic psychological need satisfaction—encompassing autonomy, competence and relatedness, and treated as a construct distinct from intrinsic motivation (Deci and Ryan, 2000)—was measured with the Basic Psychological Need Satisfaction Scale (BPNSS; Ryan and Deci, 2000b), an instrument widely used in SDT research. Each dimension was measured with five Likert-type items. Representative items included autonomy statements (e.g., “I have meaningful choices in completing assignments”), competence evaluations (e.g., “I often feel very competent when engaging in self-directed learning in this course”), and relatedness indicators (e.g., “My ideas are valued during discussions”). Confirmatory factor analysis confirmed structural validity (SMC > 0.8; Litalien et al., 2017), and the internal-consistency reliability of the subscale was Cronbach’s *α* = 0.869. Reverse-scored items controlled acquiescence bias, and cognitive interviews verified linguistic precision (Patall et al., 2019).

#### 3.3.4 Student engagement

Student engagement was assessed through four theoretically grounded dimensions (Fredricks et al., 2004), consistent with established practice for measuring engagement in technology-mediated learning (Henrie et al., 2015). Behavioural engagement measured observable effort through five items assessing attendance, persistence and task perseverance (Skinner et al., 2009). Affective engagement evaluated emotional investment using indicators of intrinsic interest and reverse-scored emotional responses. Cognitive engagement captured deep processing through metacognitive strategies and knowledge integration. Agentic engagement assessed proactive behaviours, including feedback-seeking and goal expansion (Reeve, 2013; Chiu, 2022). All constructs used five-point Likert scales contextualised to undergraduate general-education courses. Confirmatory factor analysis demonstrated construct validity (SMC > 0.5), and discriminant validity was assessed by comparing the square root of the AVE with inter-construct correlations (Hair et al., 1998). The internal-consistency reliability of the subscale was Cronbach’s *α* = 0.874.

### 3.4 Data analysis

Because the distribution of indirect effects is typically non-normal, mediation was tested with 5,000 bootstrap resamples, reporting the indirect effect, bias-corrected 95% confidence interval and significance for each group. The questionnaire was administered through online survey platforms and in-person paper forms, after which specified items were reverse-coded according to standardised protocols; participants received explicit guarantees of anonymity and confidentiality. Raw data were processed in Excel and imported into Stata 17.0, where multiple imputation addressed missing values. Structural equation modelling, incorporating path analysis, mediation and multi-group analysis, examined the relationships among extrinsic regulation, basic psychological need satisfaction and student engagement. Following Hair et al. (1998), measurement invariance was tested in three stages—configural, metric and scalar invariance—using an unconstrained model, a measurement weights model and a measurement intercepts model. To determine whether the structural paths differed significantly across groups rather than merely in magnitude, nested-model comparisons (constraining each structural path to equality and testing the change in model fit) were performed alongside the bootstrap estimates. Demographic variables were included as covariates.

**Figure 2:**
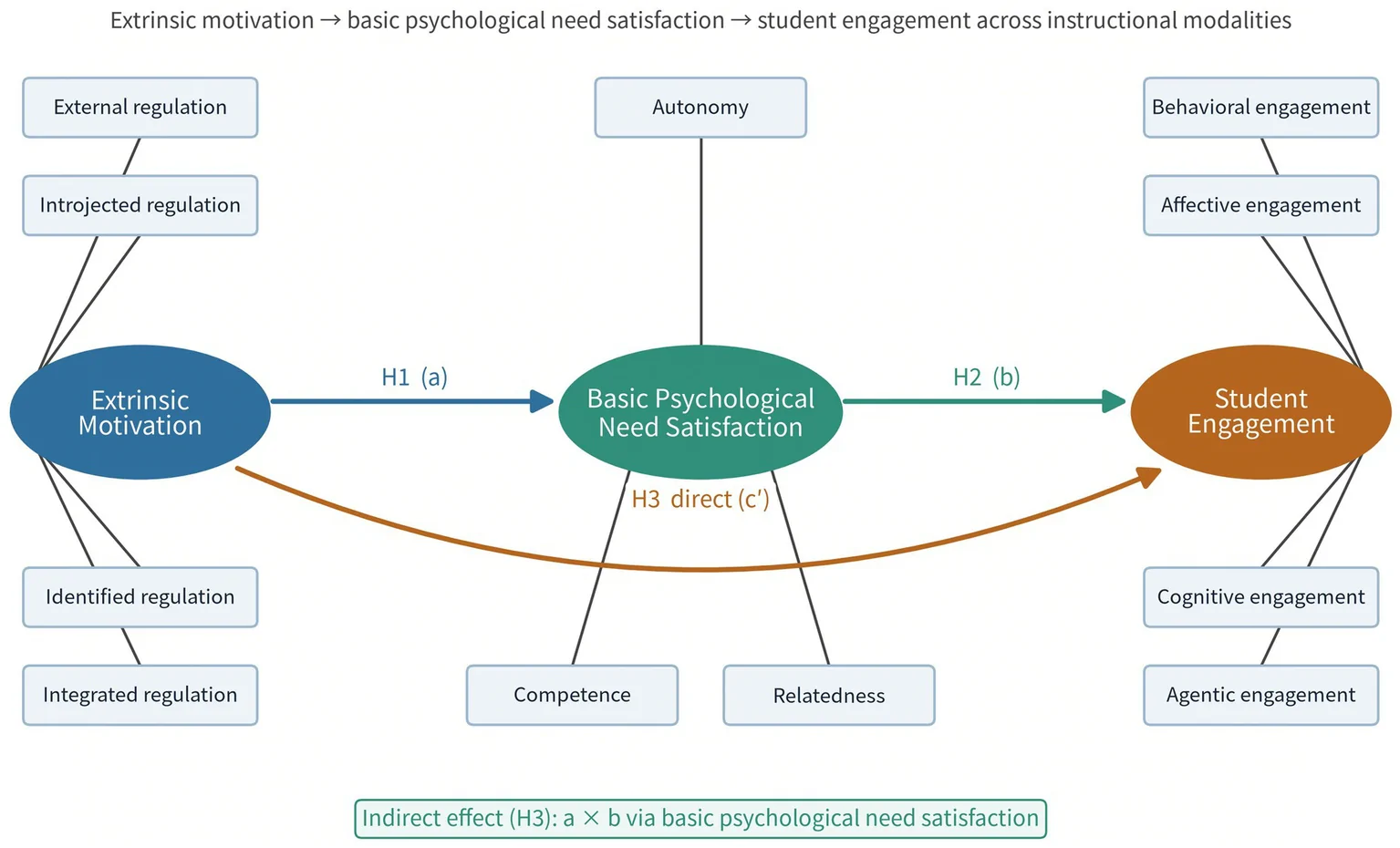
The hypothesized structural equation model.

## 4 Results

### 4.1 Descriptive statistics and prerequisite analysis

As shown in Table 3, the analyses were based on a final sample of 943 valid cases. The means of the observed indicators clustered around 3, and the medians of the binary categorical variables (Major, Gender, Hukou and Only-child) were equal to 1. The absolute skewness and kurtosis values of all observed variables fell within accepted thresholds for normality (|skewness| < 2 and |kurtosis| < 7), with a maximum absolute skewness of 0.936 and a maximum absolute kurtosis of 1.994, supporting the use of structural equation modelling. To reduce potential confounding bias, age, household registration (hukou), gender, ethnicity, only-child status, academic major, annual family income, and region were included as covariates in the model. Statistical results indicated that all three hypotheses in the structural equation model were supported regardless of whether these covariates were included. To conserve table space and maintain model parsimony, the regression estimates for these covariates are not reported in the table.

**Table 3:** Descriptive statistics of the observed variables.

Variable	*N*	Min	Max	Mean	SD	Skewness	Kurtosis
External regulation	943	1	5	2.765	1.251	0.259	1.435
Introjected regulation	943	1	5	3.032	1.257	−0.350	1.967
Identified regulation	943	1	5	3.181	1.249	−0.117	1.989
Integrated regulation	943	1	5	2.948	1.256	0.356	1.951
Autonomy	943	1	5	2.863	1.257	0.451	1.974
Competence	943	1	5	3.134	1.263	−0.157	1.936
Relatedness	943	1	5	3.033	1.261	−0.465	1.939
Behavioral engagement	943	1	5	2.849	1.266	0.153	1.950
Affective engagement	943	1	5	3.072	1.246	−0.932	1.964
Cognitive engagement	943	1	5	2.998	1.254	−0.936	1.930
Agentic engagement	943	1	5	2.962	1.255	0.466	1.994

As shown in Table 2, the squared multiple correlations (SMCs) of the observed indicators were generally above 0.5 (range 0.506–0.645). The average variance extracted (AVE) for each latent variable exceeded 0.7, indicating adequate convergent validity for the Extrinsic Motivation, Basic Psychological Need Satisfaction (BPNS) and Student Engagement constructs. Composite reliability ranged from 0.914 to 0.925, exceeding the recommended threshold of 0.70. Standardised factor loadings ranged from 0.834 to 0.895 for extrinsic motivation, from 0.715 to 0.754 for BPNS, and from 0.831 to 0.865 for student engagement. Cronbach’s alpha coefficients ranged from 0.869 to 0.891, exceeding the 0.80 threshold for good reliability, and all latent variables were positively and significantly correlated (*p* < 0.01).

As shown in Table 4, the goodness-of-fit indices for the unconstrained (configural), measurement weights (metric) and measurement intercepts (scalar) models indicated acceptable to good fit. The *χ^2^*/df values ranged from 3.118 to 3.994 (below 5.0), RMSEA from 0.034 to 0.043 (below 0.05), and SRMR from 0.025 to 0.032 (below 0.05); CFI and TLI exceeded 0.90, and PNFI and PCFI exceeded 0.5, satisfying the assumptions for subsequent path analysis.

**Table 4:** Multi-group model fit and measurement invariance.

Model	*χ^2^*/df	RMSEA	SRMR	CFI	TLI	PNFI	AIC
Unconstrained (configural)	439.848/123	0.039	0.028	0.923	0.921	0.612	358.688
Measurement weights (metric)	433.402/139	0.034	0.025	0.949	0.947	0.518	338.097
Measurement intercepts (scalar)	643.034/161	0.043	0.032	0.915	0.913	0.726	380.148

### 4.2 Pre-test analysis of student engagement

Before the intervention, a one-way ANOVA tested whether pre-test engagement differed across the three teaching groups (1 = face-to-face, 2 = online, 3 = blended); preliminary checks indicated approximate normality. As shown in Table 5, no significant between-group differences were found for behavioural (Prob > *F* = 0.771), affective (0.420), cognitive (0.784) or agentic (0.888) engagement. The three groups were equivalent at baseline, supporting the causal interpretation of the instructional intervention.

**Table 5:** One-way ANOVA of pre-test student engagement across groups.

Variables	Source	SS	df	MS	*F*	Prob > *F*
Behavioral engagement_pre	Between groups	0.802	2	0.401	0.260	0.771
	Within groups	1448.165	940	1.541		
	Total	1448.967	942	1.538		
	chi2(2) =	1.231	Prob>chi2 =	0.540		
Affective engagement_pre	Between groups	2.692	2	1.346	0.870	0.420
	Within groups	1456.719	940	1.550		
	Total	1459.410	942	1.549		
	chi2(2) =	1.028	Prob>chi2 =	0.598		
Cognitive engagement_pre	Between groups	0.771	2	0.385	0.240	0.784
	Within groups	1488.616	940	1.584		
	Total	1489.387	942	1.581		
	chi2(2) =	4.157	Prob>chi2 =	0.125		
Agentic engagement_pre	Between groups	0.368	2	0.184	0.120	0.888
	Within groups	1449.628	940	1.542		
	Total	1449.996	942	1.539		
	chi2(2) =	0.885	Prob>chi2 =	0.642		

### 4.3 Measurement invariance and model stability

As shown in Table 4, the unconstrained model yielded *χ^2^* = 439.848, df = 123, *p* = 0.665, *χ^2^*/df = 3.576, indicating configural invariance; the measurement weights model yielded *χ^2^* = 433.402, df = 139, *p* = 0.885, *χ^2^*/df = 3.118, indicating metric invariance; and the measurement intercepts model yielded *χ^2^* = 643.034, df = 161, *p* = 0.093, *χ^2^*/df = 3.994, indicating scalar invariance. Composite reliability exceeded 0.9 in each model. The measurement weights model produced the lowest AIC (338.097), followed by the unconstrained (358.688) and measurement intercepts (380.148) models; it was therefore preferred, and the cross-group path and mediation results below are based on it. Nested-model comparisons then tested the structural paths formally: each structural path was constrained to equality across the three groups in turn, and the resulting change in model fit (Δ*χ^2^* for Δdf = 2) was evaluated. Constraining the a path (extrinsic motivation → basic psychological need satisfaction) and the b path (need satisfaction → engagement) to equality did not significantly worsen fit (all Δ*χ^2^ p* > 0.05), indicating that these paths were invariant across modalities; constraining the direct c′ path did significantly worsen fit (Δ*χ^2^ p* < 0.05), confirming that the direct path differed across groups and was non-significant only in the online group.

### 4.4 Effects of extrinsic motivation on basic psychological need satisfaction

Path analysis estimated standardised coefficients and squared multiple correlations (*R^2^*). As shown in Table 6, extrinsic motivation positively and significantly predicted need satisfaction in measurement weights model. In the face-to-face group, *β* = 0.569 (*p* < 0.01), *R^2^* (*β^2^*) = 0.324; in the blended group, *β* = 0.577 (*p* < 0.01), *R^2^* (*β^2^*) = 0.333; and in the online group, *β* = 0.561 (*p* < 0.01), *R^2^* (*β^2^*) = 0.315. Extrinsic motivation thus explained 31.5–33.3% of the variance in BPNS. H1 was supported (Figure 2).

**Table 6:** Standardized regression weights in multiple-group.

Models	Teaching groups	Face to face	Blended	Online
	Path direction	Standardized regression weights	Standardized regression weights	Standardized regression weights
Unconstrained model (Configural invariance)	BPNS ← Extrinsic motivation	0.569^***^ (0.076)	0.572^***^ (0.078)	0.561^***^ (0.082)
	Student engagement ← Extrinsic motivation	0.138^**^ (0.142)	0.167^**^ (0.087)	0.18^**^ (0.085)
	Student engagement ← BPNS	0.548^***^ (0.079)	0.54^***^ (0.114)	0.533^***^ (0.098)
	Behavioral engagement ← Student engagement	0.751^***^ (0.073)	0.719^***^ (0.074)	0.726^***^ (0.083)
	Affective engagement ← Student engagement	0.746^***^ (0.073)	0.742^***^ (0.093)	0.714^***^ (0.099)
	Cognitive engagement ← Student engagement	0.704^***^ (0.073)	0.723^***^ (0.089)	0.635^***^ (0.098)
	Agentic engagement ← Student engagement	0.777^***^ (0.074)	0.734^***^ (0.092)	0.727^***^ (0.104)
	External regulation ← Extrinsic motivation	0.715^***^ (0.049)	0.691^***^ (0.084)	0.727^***^ (0.089)
	Introjected regulation ← Extrinsic motivation	0.75^***^ (0.082)	0.79^***^ (0.098)	0.782^***^ (0.095)
	Identified regulation ← Extrinsic motivation	0.798^***^ (0.082)	0.79^***^ (0.099)	0.789*** (0.095)
	Integrated regulation ← Extrinsic motivation	0.738^***^ (0.081)	0.73^***^ (0.096)	0.727^***^ (0.095)
	Relatedness ← BPNS	0.724^***^ (0.077)	0.764^***^ (0.126)	0.7^***^ (0.094)
	Competences ← BPNS	0.765^***^ (0.079)	0.751^***^ (0.122)	0.746^***^ (0.099)
	Autonomys ← BPNS	0.74^***^ (0.064)	0.653^***^ (0.079)	0.758^***^ (0.084)
Measurement weights (Metric invariance)	BPNS ← Extrinsic motivation	0.569^***^ (0.066)	0.577^***^ (0.07)	0.561^***^ (0.073)
	Student engagement ← Extrinsic motivation	0.165^**^ (0.075)	0.262^**^ (0.073)	0.122 (0.084)
	Student engagement ← BPNS	0.549^***^ (0.078)	0.545^***^ (0.095)	0.535^***^ (0.093)
	Behavioral engagement ← Student engagement	0.756^***^ (0.044)	0.717^***^ (0.053)	0.715^***^ (0.089)
	Affective engagement ← Student engagement	0.749^***^ (0.05)	0.736^***^ (0.05)	0.719^***^ (0.05)
	Cognitive engagement ← Student engagement	0.707^***^ (0.049)	0.704^***^ (0.049)	0.658^***^ (0.049)
	Agentic engagement ← Student engagement	0.768^***^ (0.05)	0.756^***^ (0.083)	0.716^***^ (0.05)
	External regulation ← Extrinsic motivation	0.719^***^ (0.074)	0.696 (0.084)	0.716^***^ (0.088)
	Introjected regulation ← Extrinsic motivation	0.756^***^ (0.052)	0.784^***^ (0.052)	0.78^***^ (0.052)
	Identified regulation ← Extrinsic motivation	0.795^***^ (0.053)	0.787^***^ (0.053)	0.796^***^ (0.053)
	Integrated regulation ← Extrinsic motivation	0.731^***^ (0.052)	0.738^***^ (0.052)	0.73^***^ (0.052)
	Relatedness ← BPNS	0.736^***^ (0.055)	0.723^***^ (0.055)	0.719^***^ (0.055)
	Competences ← BPNS	0.766^***^ (0.055)	0.746^***^ (0.055)	0.748^***^ (0.055)
	Autonomys ← BPNS	0.725 (0.439)	0.699^***^ (0.063)	0.737^***^ (0.083)
Measurement intercepts (Scalar invariance)	BPNS ← Extrinsic motivation	0.567^***^ (0.066)	0.569^***^ (0.071)	0.564^***^ (0.073)
	Student engagement ← Extrinsic motivation	0.152^**^ (0.076)	0.112^**^ (0.074)	0.176^**^ (0.089)
	Student engagement ← BPNS	0.542^***^ (0.078)	0.438^***^ (0.099)	0.497^***^ (0.097)
	Behavioral engagement ← Student engagement	0.757^***^ (0.073)	0.732^***^ (0.073)	0.723^***^ (0.088)
	Affective engagement ← Student engagement	0.747^***^ (0.049)	0.75^***^ (0.049)	0.727^***^ (0.049)
	Cognitive engagement ← Student engagement	0.707^***^ (0.048)	0.72^***^ (0.048)	0.669^***^ (0.048)
	Agentic engagement ← Student engagement	0.769^***^ (0.049)	0.773^***^ (0.049)	0.724^***^ (0.049)
	External regulation ← Extrinsic motivation	0.718^***^ (0.439)	0.695^***^ (0.083)	0.716^***^ (0.087)
	Introjected regulation ← Extrinsic motivation	0.756^***^ (0.052)	0.784^***^ (0.052)	0.78^***^ (0.052)
	Identified regulation ← Extrinsic motivation	0.796^***^ (0.053)	0.787^***^ (0.053)	0.796^***^ (0.053)
	Integrated regulation ← Extrinsic motivation	0.731^***^ (0.052)	0.738^***^ (0.052)	0.73^***^ (0.052)
	Relatedness ← BPNS	0.736^***^ (0.055)	0.726^***^ (0.055)	0.712^***^ (0.055)
	Competences ← BPNS	0.766^***^ (0.056)	0.753^***^ (0.056)	0.752^***^ (0.056)
	Autonomys ← BPNS	0.724^***^ (0.064)	0.702^***^ (0.054)	0.739^***^ (0.084)

^*^p < 0.1; ^**^p < 0.05; and ^***^p < 0.01.

Standardised coefficients; ^**^p < 0.05, ^***^p < 0.01.

### 4.5 Effects of basic psychological need satisfaction on student engagement

As shown in Table 6, BPNS positively and significantly predicted student engagement in measurement weights model. In the face-to-face group, *β* = 0.549 (*p* < 0.01), *R^2^* = 0.395; in the blended group, *β* = 0.545 (*p* < 0.01), *R^2^* = 0.362; and in the online group, *β* = 0.535 (*p* < 0.01), *R^2^* = 0.401. Extrinsic motivation and need satisfaction jointly explained 36.2–40.1% of the variance in engagement. H2 was supported.

### 4.6 Mediated effects of extrinsic motivation on student engagement

The direct, indirect and total effects of extrinsic motivation on student engagement, estimated by bootstrap resampling (5,000 samples), are reported in Table 7. In the face-to-face group, the direct effect was 0.165 (*p* < 0.05) and the indirect effect 0.312 (*p* < 0.01), bias-corrected 95% CI excluding zero, giving a total effect of 0.477; the indirect effect was approximately 1.89 times the direct effect and accounted for 65.41% of the total. In the online group, the direct effect was 0.122 and non-significant (*p* > 0.10), whereas the indirect effect was 0.3 (*p* < 0.01),95% CI excluding zero, accounting for 71.09% of the total effect (0.422). In the blended group, the direct effect was 0.262 (*p* < 0.05) and the indirect effect 0.314 (*p* < 0.01), 95% CI excluding zero, giving a total effect of 0.576; the indirect effect was approximately 1.20 times the direct effect and accounted for 54.51% of the total. H3 was supported. The multi-group analysis therefore indicates that, in fully online instruction, extrinsic motivation was not directly associated with engagement once need satisfaction was considered; it was associated with engagement only indirectly through the satisfaction of autonomy, competence and relatedness.

**Table 7:** Bootstrap direct, indirect and total effects on student engagement.

Group	Direct effect	Indirect effect (95% CI)	Bootstrap 95% conf. interval	Total effect	Indirect/total
Face-to-face	0.165^**^	0.312^***^	0.206–0.423	0.477	65.41%
Online	0.122	0.30^***^	0.194–0.408	0.422	71.09%
Blended	0.262^**^	0.314^***^	0.208–0.422	0.576	54.51%

The fourth column of the table reports the bias-corrected 95% confidence intervals for the indirect effects estimated using the bootstrap method (5,000 resamples). All confidence intervals exclude zero; ^*^p < 0.1, ^*^p < 0.05, ^***^p < 0.01.

## 5 Discussion

### 5.1 Extrinsic motivation as a need-supportive resource across modalities

The first notable finding is that extrinsic motivation was positively and comparably related to basic psychological need satisfaction across the three modalities (*β* = 0.561–0.577), providing consistent support for H1. This result challenges the assumption that extrinsic motivation is uniformly detrimental to autonomous functioning. Within SDT, extrinsic motivation is not a single construct but a set of regulations that vary in relative autonomy, from external and introjected regulation to identified and integrated regulation (Ryan and Deci, 2000a). The positive association observed here indicates that, in these general-education courses, the extrinsic regulations measured were largely internalised rather than experienced as purely coercive, and therefore functioned as a resource for autonomy, competence and relatedness. This interpretation is consistent with meta-analytic evidence that the motivational consequences of rewards depend on how they are communicated: tangible, controlling rewards tend to undermine intrinsic motivation, whereas non-controlling recognition and informational feedback can sustain it (Deci et al., 1999). More recent work reinforces this distinction, showing that financial rewards relate negatively to intrinsic task motivation whereas recognition and social rewards relate positively, because the latter convey competence and acknowledge personal value (Malek et al., 2020), and that rewards operate through situational extrinsic motivation (Yoon et al., 2015). With need satisfaction explaining roughly one third of its variance (*R^2^* = 0.315–0.333), extrinsic motivation is a meaningful but not exhaustive source of psychological nourishment. The similarity of the path coefficients across modalities further suggests that this capacity is robust to instructional context: incentives and feedback appear to satisfy needs when perceived as informational and autonomy-supportive rather than controlling (Ryan and Deci, 2020). This pattern aligns with evidence that need-supportive teacher and digital practices predict motivation and engagement in both online and AI-assisted settings (Chiu, 2022; Chiu et al., 2024).

### 5.2 Need satisfaction as the proximal driver of engagement

The second finding is that basic psychological need satisfaction consistently predicted student engagement (*β* = 0.535–0.549), supporting H2 and confirming the status of needs as the proximal motivational source of behavioural, emotional, cognitive and agentic investment. This result is consistent with the multidimensional view of engagement, which treats these components as distinct yet functionally coordinated forms of academic investment rather than interchangeable indicators (Fredricks et al., 2004; Reeve et al., 2025). Across all three modalities, students who experienced greater autonomy, competence and relatedness engaged more deeply and more durably, as SDT predicts when social contexts supply the nutrients for optimal functioning (Ryan and Deci, 2000b; Niemiec and Ryan, 2009). The explanatory power of the structural equations (*R^2^* = 0.362–0.401) indicates that need satisfaction is a substantial driver of engagement regardless of instructional mode. This converges with evidence from online learning during the COVID-19 pandemic, where need satisfaction predicted engagement and relatedness support was especially decisive (Chiu, 2022), and with studies of AI-based chatbots and AI-assisted learning, in which perceived support for competence and autonomy translated into sustained motivated behaviour (Chiu et al., 2024; Xu and Zhi, 2026; Wu and Yu, 2024). In substantive terms, engagement is not secured by technology or incentives alone; it follows from the degree to which an environment—whether a classroom, a MOOC or a SPOC—makes students feel volitional, effective and connected, consistent with the distinction between engagement and motivation or achievement (Wong and Liem, 2022; Raes et al., 2019).

### 5.3 The mediated pathway and its qualification by modality

The third and most important finding is that need satisfaction significantly mediated the extrinsic motivation–engagement relationship in every modality, supporting H3, while the modality comparison revealed a theoretically meaningful difference. The indirect effect accounted for 54.51% of the total effect in the blended group, 65.41% in the face-to-face group and 71.09% in the online group, indicating that extrinsic motivation sustains engagement chiefly through the internalisation pathway. This mediation logic is consistent with prior evidence that teacher and digital support operate indirectly through psychological and self-regulatory processes, such as self-efficacy, achievement goals, motivated learning behaviour and engagement itself (Liu et al., 2023; Li et al., 2024; Kok et al., 2025). The decisive contrast concerns the direct path: it was significant in the blended (0.262) and face-to-face (0.165) groups but non-significant in the online group, where only the indirect effect (0.3) was significant. Because the structural relationships rest on self-reported perceptions, these associations should be interpreted as consistent with, rather than definitive proof of, the SDT causal sequence. The pattern is nonetheless theoretically coherent: online learning structurally weakens social presence and relatedness, and needs must be deliberately engineered through digital design (Chiu, 2022; Raes et al., 2020). The blended group produced the largest indirect effect (0.314), supporting the argument that integrating face-to-face relatedness with flexible, self-regulated online learning can strengthen the internalisation mechanism, whereas flexibility benefits students only when accompanied by coherent pedagogy rather than mere technological access (van der Rijst et al., 2023; Howell, 2022). Practically, online instructors cannot rely on grades or deadlines alone; they must actively build interaction, feedback and belonging so that external incentives are internalised. Together, these results qualify the apparent universality of the SDT sequence: the mediating mechanism holds across modalities, but its magnitude and the availability of a direct motivational route depend on the social presence the modality affords.

## 6 Conclusion

### 6.1 Theoretical contributions

Drawing on self-determination theory, this study contributes to the literature in three ways. First, it treats extrinsic motivation as a differentiated set of regulations rather than a unidimensional, uniformly detrimental construct, and demonstrates that it is positively associated with basic psychological need satisfaction in higher education. Second, it positions need satisfaction as the central mechanism linking motivation to behavioural, emotional, cognitive and agentic engagement, with the indirect pathway accounting for roughly two-thirds of the total effect across learning environments. Third, by establishing configural, metric and scalar invariance and conducting multi-group comparisons, the study shows that this mediation is robust across face-to-face, online and blended instruction but qualified by modality: the direct motivation–engagement path disappears in fully online settings. The findings therefore integrate SDT with research on instructional modalities and AI-enhanced learning, clarifying when and why extrinsic motivation is internalised into sustained engagement.

### 6.2 Practical implications

The findings offer practical guidance for higher-education teaching. Instructors and course designers should use rewards, grades and feedback informationally rather than controllingly, pairing them with meaningful choice, appropriately challenging tasks and opportunities for relatedness. Online and blended courses merit particular attention: because extrinsic motivation was not directly associated with engagement in fully online settings, digital platforms should deliberately engineer interaction, timely feedback and social presence, using AI tools to scaffold competence and autonomy while preserving the human support that satisfies relatedness. Aligning teacher and digital support across modalities is therefore essential for maintaining student engagement.

## 7 Limitations and future directions

Several limitations should be acknowledged. First, the data were drawn from undergraduates at one university using convenience and quota sampling, which limits generalisability to other disciplines, institutions and cultural contexts. Second, although the pre-test and quasi-experimental design strengthen causal plausibility, the structural relationships rest on self-reported, single-source survey data and remain vulnerable to common-method bias; longitudinal or fully randomised designs would better establish temporal and causal order. Third, the extrinsic motivation subscale relied on adapted items, and future research should validate the full OIT continuum, including intrinsic motivation. Finally, the study did not examine specific AI features or teacher behaviours in detail. Future work could combine objective trace data, qualitative interviews and cross-institutional samples to test the mechanism across diverse AI-enhanced learning environments.

## Data Availability

The raw data supporting the conclusions of this article will be made available by the authors, without undue reservation.
